# Nonsyndromic Oral Cleft in First-Degree Relatives of Patients with Acute Lymphoblastic Leukemia

**DOI:** 10.3390/dj8010023

**Published:** 2020-03-04

**Authors:** Verônica Oliveira Dias, Daniella Reis Barbosa Martelli, Maria Luiza Santos, Célia Márcia Fernandes Maia, Rodrigo Soares de Andrade, Ricardo D. Coletta, Hercílio Martelli Júnior

**Affiliations:** 1 Dental School, State University of Montes Claros, Montes Claros 39.400-000, Minas Gerais, Brazil; daniellareismartelli@yahoo.com.br (D.R.B.M.); marialuizasantos25@hotmail.com (M.L.S.); hmjunior2000@yahoo.com (H.M.J.); 2 Health Science Program, State University of Montes Claros, Montes Claros 39.400-092, Minas Gerais State, Brazil; celiamaiaop@terra.com.br; 3 Department of Oral Diagnosis, School of Dentistry, University of Campinas, Piracicaba 13.414-018, São Paulo, Brazil; rodrigosoares002@hotmail.com (R.S.d.A.); coletta@fop.unicamp.br (R.D.C.); 4 Center for Rehabilitation of Craniofacial Anomalies, Dental School, University of José Rosario Vellano, Alfenas 37.130-000, Minas Gerais State, Brazil

**Keywords:** nonsyndromic oral cleft, cancer, acute lymphoblastic leukemia

## Abstract

Multiple studies have demonstrated an association between cancer and nonsyndromic oral clefts in different populations. In this study, we assessed the occurrence of nonsyndromic oral clefts in families of patients with acute lymphoblastic leukemia (ALL, *n* = 50) and controls (*n* = 125). The parents of the patients answered a questionnaire with basic demographic information and family history of nonsyndromic oral clefts in first-degree relatives. Statistical analysis was carried out using Fisher’s exact test. In the ALL group, 22 (44%) were male and 28 (56%) were female, and the average age was 13.2 ± 12.2 years. In the control group, 64 (51.2%) were male and 65 were female and the average age was 11.3 ± 10.3 years. Two out of 50 patients (4%) with acute lymphoblastic leukemia had a positive history of nonsyndromic oral clefts, whereas there were no reported occurrences of nonsyndromic oral clefts in the control group (OR: 12.94, 95% CI: 0.61–274.6, *p* = 0.08). Despite the limited population, the frequency of nonsyndromic oral clefts was increased in the first-degree relatives of patients with acute lymphoblastic leukemia. Studies with larger samples and molecular analyses are needed to better understand the possible etiological relationship between cancer and nonsyndromic oral clefts.

## 1. Introduction

Oral clefts, representing cleft lip only, cleft lip and palate, and cleft palate only, are the most common orofacial birth defects, occurring in 1 in 500–2500 live births worldwide [1]. The prevalence in Brazil is from 0.36 to 1.54 per 1000 live births [2,3], and approximately 70% of the cases occur as a nonsyndromic form (nonsyndromic oral cleft—NSOC) [4]. NSOCs are caused by a complex interplay between environmental exposures, particularly maternal lifestyle (smoking and consumption of alcohol), diet, certain drugs and medicines, and social, occupational, and residential risk factors, along with genetic factors with influence of ancestry [1,5,6]. We recently performed a systematic review and meta-analysis to verify the genetic variants associated with NSOC susceptibility in the Brazilian population and demonstrated that variants in *IRF6*, 8q24 and *MTHFR* were associated with increased risk for nonsyndromic cleft lip with or without cleft palate, whereas rs17563 in *BMP4* showed a protective effect [7].

Approximately 6000 cases of acute lymphoblastic leukemia (ALL) are diagnosed in the United States annually, with half of the cases occurring in children and teenagers. It is the most common cancer among children and the most frequent cause of death due to cancer before the age of 20 [8,9]. In Brazil, the incidence of ALL is about 40 cases per million individuals under 15 years with a peak incidence from 2 to 5 years of age [10].

Surgery along with extensive dental treatment and other therapies is very important to correct the physical health defects related to NSOC. However, there remains a significant social, emotional and financial burden for the individuals and their families. Moreover, other long-term adverse complications are more common in patients with NSOC and their relatives than in the general population, including risk of cancer [1]. Indeed, it has been proposed that cancer and NSOC may occasionally have a common etiology. The concept is that genes acting during the normal development may also have an important role in malignancies [6]. Over the past years, epidemiological studies have investigated the association of cancer with NSOC in different populations, including those from the United States, Southeast Asia, Denmark, Latvia, India, the Netherlands, Turkey, France, Poland, Pakistan, and Brazil [11,12,13,14,15,16,17,18,19,20,21,22,23,24,25,26], and the results are controversial. Thus, the aim of this study was to determine the frequency of NSOC in families of patients with a diagnosis of ALL.

## 2. Material and Methods

### 2.1. Sample

The study included all patients with ALL (case group; *n* = 50) diagnosed and treated in a reference Public Hospital in Oncology, in Minas Gerais, Brazil, between 2016 and 2018. The control group included 125 healthy individuals, cancer-free, randomly obtained at the general clinics at the University and Hospital. The sampling was performed by convenience and the groups were matched concerning gender and age. During the interview of mothers by a trained staff, gender and age of subject, gestational age (number of weeks that the fetus had grown inside the mother’s uterus), if the child is her first-born, maternal age at conception, and family history of NSOC (cleft lip only, cleft lip and palate, or cleft palate only) in first-degree relatives (individual’s parents, siblings, and children) for both groups were obtained. All subjects enrolled in this study reside in the same geographical area (Northern Minas Gerais, Brazil). Thus, the control group presented similar demographic, ethnic and sociocultural characteristics as the ALL group. Informed consent was obtained according to the declaration of Helsinki, and the study was approved by the Research Ethics Committee of the University (1.416.786/2016) (ethics approval date: 20/02/2016).

### 2.2. Statistical Analysis

Differences in demographic and clinical characteristics between groups were assessed with *t*-test or chi-squared test. To determine the association of NSOC risk in first-degree relatives of ALL patients, Fisher’s exact test with odds ratio (OR) and a 95% confidence interval (95% CI) was used. The level of significance considered was 5% (*p* < 0.05).

## 3. Results

Demographic and clinical characteristics of subjects included in this study are depicted in Table 1. The average age was 13.2 ± 12.2 years for the cases diagnosed with ALL and 11.3 ± 10.3 years in the control group (*p* = 0.30). The proportion of males and females was 56% and 44% in the ALL group, and 48.8% and 51.2% in the control group (*p* = 0.38). There were no differences between groups regarding gestational age, first-born children, and maternal age at conception (Table 1).

In the ALL group, two (4%) patients had a positive family history of NSOC, while none of the control group subjects reported a family history of NSOC (*p* = 0.08, Table 2). With this sample size, the frequency of NSOC as 1:1000 live births, and the average Brazilian family size of 16 individuals [27], we would expect to identify approximately 0.8–2 cases of NSOC in relatives of ALL patients (50 × 16/1000) and 2–3 cases among relatives of the controls (125 × 16/1000). Thus, in the present study the frequency of NSOC was higher in the first-degree relatives of patients with ALL than in the control group.

## 4. Discussion

Cancer is a leading cause of death, and approximately 14 million new cancer cases worldwide are reported every year [28]. Carcinogenesis is a complex and heterogenous process, involving interactions among environmental exposures, lifestyle, and internal factors. In terms of internal factors, the main manifestations are genetic variations in driver genes, immune system failures and alterations in hormone secretion [29]. Given the complex heterogeneity and pathogenicity of cancer, all these risk factors appear to be reciprocal and thus required in scrutiny of disease prognosis [28].

Investigating the association of malformations with malignancies is important, as it is suggested that they might have common etiopathology. In this study, the frequency of NSOC was higher in the first-degree relatives of patients with ALL than in the control group. The occurrence of NSOC was more frequent in families with ALL than in families without the diagnosis of this pediatric cancer in India [15]. A higher risk for leukemia in relatives of children born with oral clefts was also reported [16].

Menezes and collaborators [15] showed a higher frequency of family history of cancer (colon, brain, leukemia, breast, prostate, skin, lung, and liver) in relatives of NSOC compared to a control group. A higher prevalence of cancer in family members of individuals born with NSOC was presented in a cross-sectional study with a Latvian population [17]. The risk was calculated by dividing the prevalence of cancer in the target group by the prevalence of cancer in that population. It was demonstrated that this risk is three times higher in first- and second-degree relatives and decreases to 1.5 times in third-degree relatives. On the other hand, Martelli and collaborators [24] were unable to confirm a general increase in the risk of NSOC among families of women with breast cancer.

Although multiple candidate genes and genetic loci conferring susceptibility to NSOC have been associated with cancer risk [11,12,13,14,15,16,17,18,19,20,21,22,23,24,25,26], the genetic pathways underlying cancer and birth defects remain to be identified. Interestingly, NSOC is ultimately caused by interferences in cell proliferation, differentiation, survival, adhesion and migration, all of which are known to be involved in cancer [4]. We recently demonstrated that polymorphic variants in *AXIN2* and *CDH1*, which are involved in cell adhesion and migration and are strongly related to breast and gastric cancer, are associated with NSOC, reinforcing the putative link between cancer and oral clefting [5]. A recent study revealed a significant association between oral cancer risk and variants in *GSK3β* (rs9879992) and *WNT11* (rs1533767) [30], which are also associated with NSOC risk [31]. Members of the WNT signaling pathway, including *GSK3β* and *WNT11*, control migration, polarity, and fate during embryonic development and are widely implicated in human cancers [32]. Gene networks governing cellular defenses against DNA damage were associated with the etiology of NSOC, in accordance with the idea that orofacial clefts and cancer may have overlapping etiologies [33]. However, as multifactorial diseases, besides the genetic predisposition, we should consider the role of environmental factors that may interact with the genetic risks to develop these diseases, such as smoking and alcohol, among other factors. Some limitations should be considered in the present study, including the use of self-reported information and the memory bias associated with the study design, the reason to choose the history of NSOC only in first-degree relatives, and the relatively small number of patients with ALL. Studies with larger samples and molecular analyses are needed to better understand the possible association between cancer and NSOC.

## Figures and Tables

**Table 1:** Characteristics of patients with acute lymphoblastic leukemia (ALL group) and control group.

	Control Group	ALL Group	*p*-Value
Age			
Mean ± SD (years)	11.3 ± 10.3	13.2 ± 12.2	0.30 ^a^
Gender			
Male	64 (51.2%)	22 (44%)	
Female	65 (48.8%)	28 (56%)	0.38 ^b^
Gestational age			
<37 weeks	15 (12.3%)	2 (4.2%)	
37–41 weeks	101 (82.8%)	43 (89.6%)	0.27 ^b^
≥42 weeks	6 (4.9%)	3 (6.2%)	
First-born			
Yes	50 (40%)	15 (30%)	0.21 ^b^
No	75 (60%)	35 (70%)	
Maternal Age			
<35 years	113 (90.4)	44 (88)	0.63 ^b^
≥35 years	12 (9.6)	6 (12)	

^a^*t*-test; ^b^ chi-squared test.

**Table 2:** Frequency of nonsyndromic oral cleft in first-degree relatives of patients with acute lymphoblastic leukemia (ALL).

	Control Group	ALL Group	OR	95% CI	*p*-Value
Nonsyndromic oral cleft					
No	125 (100%)	48 (96%)			
Yes	0	2 (4%)	12.94	0.61–274.6	0.08
